# Editorial: Microenvironment and therapy-resistance in leukemias

**DOI:** 10.3389/fonc.2023.1244642

**Published:** 2023-07-14

**Authors:** Rachel Friedmann, Yong-Mi Kim, Marina Konopleva

**Affiliations:** ^1^ Children’s Hospital Los Angeles, Department of Pediatrics, Division of Hematology and Oncology, University of Southern California, Keck School of Medicine, Los Angeles, CA, United States; ^2^ Department of Medicine (Oncology) and Molecular Pharmacology, Albert Einstein College of Medicine, Bronx, NY, United States

**Keywords:** acute lymphoblastic leukemia, acute myeloid leukemia, drug resistance, microenvironment, targeted therapy

The tumor microenvironment (TME) can mediate resistance of acute leukemias to chemotherapy, however few therapies targeting this niche interaction are available. This Research Topic highlights recent findings regarding “*Microenvironment and Therapy-Resistance in Leukemias*”.

In the review by Hellmich et al. the authors focus on the role of senescence in the aging bone marrow (BM) microenvironment in acute myeloid leukemia (AML) and multiple myeloma. Senescence is the irreversible arrest of stem cell proliferation. Through senescence, the senescence associated secretory phenotype (SASP) is activated, causing manipulation in growth and proliferation by pro-inflammatory cytokines, chemokines, proteases and growth factors. Though evolutionarily protective as an anti-cancer mechanism promoting repair, the system of senescence can become maladaptive, and instead create a pro-inflammatory, pro-tumor growth, and chemotherapy resistant environment. The prior publication by this group (Blood 2019) (1), demonstrated that AML cells induce a senescent phenotype in BM stromal cells (BMSCs) resulting in the secretion of SASP supporting the survival and proliferation of leukemic blasts. *In-vivo* studies demonstrated that deletion of BMSCs slowed tumor progression and prolonged animal survival. This supports the role in development of anti-senolytic agents which can selectively eliminate senescent cells and reduce chemoresistance.

In the review by Tabe et al., the authors present the role of fatty acid metabolism as a targetable mechanism of AML chemoresistance in adults whose BM content is progressively replaced by adipocytes. Oxidative phosphorylation (OXPHOS) is utilized by Leukemic stem cells (LSCs) as an energy source to promote growth and survival of the tumor cells. The authors describe the potential for combinatorial regiments utilizing fatty acid oxidation (FAO) inhibitors to target the critical energy source for the chemo-resistant leukemia cells and LSCs. FAO produces twice as much ATP per mole compared to the oxidation of glucose, making it a more effective energy source. LSCs obtained from relapsed AML patients acquire a compensatory ability to overcome the loss of amino acid metabolism (targeted by chemotherapies) by increasing FAO. While FAO inhibition sounds promising, tumor cells have several mechanisms for adapting to nutrient deprivation. Therefore, FAO alone is unlikely to be useful; rather combination with chemotherapy or targeted therapies have potential for synergistic effect.

The review by Nehrbas et al. focuses on the role of Extracellular Vesicles (EVs) used to mediate crosstalk between AML cells and the BM microenvironment, which facilitates AML chemoresistance through the transport of protein, RNA, and DNA. EVs are membrane-bound secreted particles containing a variety of nucleic acid/protein material. Exosomes, an EV subtype, can traffic proteins, lipids, mRNAs, multiple types of non-coding RNAs, DNAs and chemokines/cytokines. Co-culture experiments showed that chemo-resistant AML cells could induce chemoresistance in chemo-sensitive AML cells by upregulating the anti-apoptotic protein BCL-2. EV-mediated transfer of miRNAs is known to increase chemoresistance in several cancer types. Specific microRNAs are described as inducing resistance through tyrosine kinase inhibitor resistance and overexpression of drug efflux pump multidrug resistance protein 1(MRP-1). EVs also mediate resistance to immunotherapy reducing the efficacy of adoptive Natural Killer (NK) cell therapy cells through transport of inhibitory ligands. AML cells release exosomes containing programmed death-receptor ligand 1 (PD-L1) causing T-cell suppression. It also sequesters the anti-PDL-1 antibodies leading to checkpoint inhibitor resistance. Further characterization of EV cargo, mechanisms of downstream chemoresistance and ultimately development of novel approaches to control EV biogenesis or uptake will be a potential target to improve treatment outcomes in leukemia.

Bhatnagar and Garzon review the role of MicroRNAs (miRs), short non-coding RNAs, in AML as a prognostic factor as well as therapeutic target. Dysregulated miRs can function as oncogenes or tumor suppressors, and the same miR can function as a tumor suppressor or oncogene depending on its context. The review focuses on potential clinical applications of miRs in adult AML and discusses unique miR signatures in specific AML subtypes. Though several miRs have been identified as promising targets in AML, clinical trials have not been conducted. The limiting factors include off-target effects, toxicity, and specific delivery to blasts.

Cancilla et al. review the chemokine receptor CXCR4 and its interaction with CXCL12 as a key player in the survival and migration of malignant stem cells to the BM. High expression of CXCR4 on AML and ALL blasts has been identified as a predictor of poor prognosis. Given its role in retaining AML blasts in the BM, inhibiting CXCR4 can disrupt its pro-survival signaling, induce cytotoxicity and mobilize the leukemia cells from the marrow into the vasculature where they would be more susceptible to chemotherapy. The data review the four categories of CXCR4 targeting drugs, including small molecule CXCR4 agonists, peptide-like CXCR4 antagonists, antibodies to CXCR4, and CXCL12 antagonists. The use of CXCR4 inhibitors in hematopoietic transplant myeloablation, enhancement of donor engraftment post-transplant, and the combination with checkpoint inhibitors is described.

Kim et al. reviews the role of cadherins, selectins, and integrins in cell adhesion mediated drug resistance (CAM-DR), and the results of clinical trials targeting these molecules. CAM-DR has been demonstrated in many subsets of leukemia including B- and T- acute lymphoblastic leukemia. Cell adhesion molecules (CAMs) not only recognize ligands for binding but also initiate the intracellular signaling pathways that are associated with cell proliferation, survival, and drug resistance upon binding to their ligands.

Epperly et al. review the immunosuppressive AML microenvironment as an underlying mechanism for the failure of chimeric antigen receptor T (CAR-T) cell therapy in AML. The review addresses the various cell types in the AML microenvironment which can lead to T-cell exhaustion, including AML blasts, myeloid-derived suppressor cells (MDSCs), regulatory T cells (Tregs), macrophages, and dendritic cells. Other mechanisms for immune evasion are explored such as the downregulated expression of major histocompatibility complex class I and II, the increased expression of inhibitory ligands as well as structural components of the BM and metabolic alterations including amino acid concentrations. This review sheds light on ways to enhance the AML response to CAR-T cells through targeting the immunosuppressive and anti-inflammatory aspects of the microenvironment.

In summary, this Research Topic of outstanding articles illustrates the biological and therapeutic significance of the microenvironment of therapy-resistant leukemias.

## Author contributions

MK and RF wrote the first draft of the manuscript. All authors contributed to manuscript revision, read, and approved the submitted version.
